# Examining workload variations among different surgical team roles, specialties, and techniques: a multicenter cross-sectional descriptive study

**DOI:** 10.1186/s13741-023-00356-6

**Published:** 2024-01-02

**Authors:** Sepideh Totonchilar, Akram Aarabi, Naeimeh Eftekhari, Masoumeh Mohammadi

**Affiliations:** 1 Ardabil University of Medical Science, Ardabil, Iran; 2 Isfahan University of Medical Sciences, Isfahan, Iran

**Keywords:** Workload, Surgery task load index, Surgical team

## Abstract

**Background:**

A high workload may negatively impact the surgical team's performance and jeopardize patient safety. The aim of this study was to measure the workload of the surgical team across different surgical roles, specialties, and techniques in several hospitals.

**Methods:**

This cross-sectional multicenter study was performed in the operating rooms of eight teaching hospitals affiliated with Isfahan University of Medical Sciences, Iran. At the conclusion of each surgical procedure, all members of the surgical team completed the Surgery Task Load Index (SURG-TLX) questionnaire to assess workload levels. Descriptive statistics, analysis of variance (ANOVA), and Pearson correlations, were performed to compare surgical roles, specialties, techniques, and surgical time on workload overall and by subscale.

**Results:**

A total of 409 workload questionnaires were obtained from 76 surgical teams or cases, involving 346 surgical team members. The total workload among all participants was 32.41 ± 17.21. Surgical complexity, physical demands, and mental demands were the highest workload subscales and distraction was the lowest workload subscale. Cardiovascular specialty had a higher workload compared to other specialties. Open techniques resulted in a higher workload compared to minimally invasive techniques. Surgical technologists who act in both the role of circulating and scrub nurse (C&Ss) experienced the highest workload, followed by surgical residents and surgeons.

**Conclusions:**

The results of the study showed that the workload for some members of the surgical team is disproportionately high and is influenced by factors such as specialty, technique, role, and surgical duration. By knowing the distribution of workload among the members of the surgical team, efforts can be made to optimize the team members’ workload.

## Introduction

Clinical work in the operating room is challenging because it is complex, dynamic, and often time- and resource-constrained (Göras et al. 2020). This complex structure of the operating room leads to a high workload for the surgical team (Wallston et al. 2014). The workload is a multidimensional construct, defined as the relationship between the demands of a job, the conditions under which the job is performed, and the individual's abilities, skills, behavior, and perception (DiDomenico and Nussbaum 2008; Carswell et al. 2005; Longo 2018). As surgeries become more complex and require more technology, the physical and mental demands on surgeons and their teams will increase. A high workload can compromise the performance of the surgical team, as well as patient safety (Garcia et al. 2019; Weigl et al. 2016). In a study by Suliburk et al. (2019), 188 adverse events were reported over 6 months, and 51.6% of all human performance deficiencies leading to these events were due to cognitive errors (Suliburk et al. 2019). This highlights the importance of managing cognitive workload in surgery to avoid errors and adverse outcomes. Teams in a work environment, influenced by various performance-shaping factors (PSFs), work together toward a common goal. These PSFs (such as health, mental state, and cognitive ability) can affect workload (Wallston et al. 2014). A shared mental model, which represents the collective understanding of a surgical team’s collaborative efforts, is a significant factor that affects workload. Uneven workload distribution among surgical team members and a lack of shared understanding of surgical procedure difficulty can lead to conflicts and surgical errors (Nikolai 2020; Jonker et al. 2011). Studies have shown that a shared mental model among surgical team members can improve teamwork and outcomes for patients (Nakarada-Kordic et al. 2016; Wilson 2019). Lowndes et al. study (2019) showed that teamwork in the operating room requires improvement and balancing workloads between the different roles of the surgical team (Lowndes et al. 2019). The study of mental and physical workload in surgery helps to understand the current and future needs of the work system (Wallston et al. 2014; Rieger et al. 2015), training and team needs (Weinger et al. 2004), stress, and burnout (Rieger et al. 2014). Additionally, operating room leaders have a responsibility to ensure the delivery of safe, cost-effective, quality patient care while maintaining team satisfaction with the work environment (Walters and Webb 2017).

The studies that have been done to improve the surgical workload in the operating room have mainly focused on the surgeon's needs. Moreover, previous studies have not thoroughly investigated the measurement of surgical team workload across various institutions and specialties, considering the diverse work system characteristics involved (Lowndes et al. 2019; Niichel et al. 2019; Yu et al. 2016).

Assessing surgical team workload enables healthcare professionals to identify high workload areas and enhance patient safety by reducing workload. To achieve optimal workload among surgical team members, it is crucial to measure and compare workload across various surgical roles, techniques, specialties, and healthcare centers.

## Materials and methods

### Design and setting

In this cross-sectional multicenter study, data were collected from September 2022 to April 2023 in the operating rooms of eight teaching hospitals affiliated with Isfahan University of Medical Sciences, Iran. The studied hospitals were Alzahra, Kashani, Beheshti, Feyz, Shahid Chamran, Isabn-e-Maryam, Emam Hossein, and Imam Musa Kazim. The study data were collected using nonrandom or convenience sampling with quotas, which were proportional to the number of monthly surgeries at each center.

### Data collection

At the end of each surgery, participants completed paper-based questionnaires that included demographic information and the Surgery Task Load Index (SURG-TLX) to assess perceived workload. The SURG-TLX questionnaire was developed and validated by Wilson et al. (2011) (Wilson et al. 2011). Heidarimoghadam et al. (2022) demonstrated acceptable reliability and validity of the SURG-TLX questionnaire in Iranian culture (Heidarimoghadam et al. 2022). The SURG-TLX is a multidimensional instrument designed to assess the workload of surgical team members. It calculates an overall workload score by combining six subscales. The specific dimensions for the SURG-TLX were formulated and defined as follows:

- 1. Mental demands: How mentally fatiguing was the procedure?
- 2. Physical demands: How physically fatiguing was the procedure?
- 3. Temporal demands: How hurried or rushed was the pace of the procedure?
- 4. Task complexity: How complex was the procedure?
- 5. Situational stress: How anxious did you feel while performing the procedure?
- 6. Distractions: How distracting was the operating environment?

The total possible score on the SURG-TLX ranges from 0 (very low) to 100 (very high). An additional file shows this in more detail (see Additional file 1). While the precise threshold for a negative impact on human performance is still debated, research suggests that cognitive load scores exceeding 50–55 may be linked to increased performance errors (Yu et al. 2016; Mazur et al. 2014; Mazur et al. 2013). We used 50 as a cutoff to separate lower vs. higher perceived load for any SURG-TLX subscales. The researchers collected data on several factors for each surgical case, including procedure type, surgical technique, surgical specialty, and surgical duration (from the time the patient entered the room until the patient left the room). The inclusion criteria for study participation included willingness to participate in the study, absence of clear anxiety and depression symptoms, and non-use of psychotropic medications according to self-reporting in the questionnaire. The exclusion criterion was incomplete questionnaire completion.

### Statistical analysis

Data were analyzed using two descriptive and analytical methods using SPSS version 16 software. Descriptive statistics were used to describe the surgical procedures and demographic characteristics of the participants. We examined the effects of surgical specialty, surgical technique, and team role on workload using analysis of variance (ANOVA). Post hoc tests for multiple comparisons were performed with Bonferroni correction. The effect of surgical time on perceived workload was analyzed with Pearson correlation. All statistical tests were performed at a significance level of < 0.05.

### Ethical considerations

This study was approved by the Research Ethics Committee of Isfahan University of Medical Sciences, Isfahan, Iran (code: IR.MUI.NUREMA.REC.1401.074). Written informed consent was obtained from each participant before participation in the study. The purpose of the study was explained to them, and the research team answered any questions the participants might have. They were assured that their participation in this study was completely voluntary.

## Results

A total of 409 workload questionnaires were obtained from 76 surgical teams or cases, involving 346 surgical team members from eight hospitals. Table 1 shows the abbreviated terms and responsibilities of the surgical team members. (page 17). Among the participants, 212 (51.8%) were women. In each surgery, there was an average of five surgical team members who completed the questionnaire. Table 2 presents the surgical duration and the number of participants across various surgical roles, categorized by different specialties and surgical techniques.

**Table 1:** Responsibilities by surgical team member roles

Roles	Responsibilities
Surge	Surgeon: performs and supervises the procedure
SR^a^	Surgical resident: the duties range from observation to assisting the surgeon during surgery
SN	Scrub nurse: ensuring the availability of surgical instruments and delivering them to the surgeon, counting surgical items, and assisting the surgeon
CN	Circulating nurse: checking the sterility of surgical items, opening instruments for the sterile field, counting surgical items, filling out paperwork
Anes	Anesthesiologist: supervises and administers anesthesia
AR^a^	Anesthesia resident: the duties of an anesthesia resident range from observation to assisting the Anesthesiologist
NA	Nurse anesthetist: The nurse anesthetist receives clear and unambiguous instructions from the anesthesiologist and assists the anesthesiologist before, during, and after surgery.
C&Ss	Both circulating and scrub nurse: a surgical technologist who was both a circular nurse and a scrub nurse during the same cardiac surgery. These surgical technologists were only changed intraoperatively during long cardiac surgeries.

^a^Resident: a surgical or anesthesia trainee with one to 5 years of postgraduate experience

**Table 2:** Description of surgery duration and different surgical roles, specialties, and techniques

	Team	Time (min) Mean±SD	Surg	Anes	SR	AR	SN	CN	NA	C&S	Total
**Specialty**											
General surgery	6	183.33 ± 127.06	6	3	4	2	5	5	6	0	31
Gynecology	4	117.5 ± 76.3	3	2	7	2	3	4	4	0	25
Urology	6	130 ± 110.4	3	5	6	1	6	6	6	0	33
ENT	10	107.5 ± 92.44	6	3	13	5	8	10	9	0	54
Ophthalmology	9	61.11 ± 23.55	6	5	6	1	10	9	9	0	46
Neurology	4	231.25 ± 25.28	4	2	6	4	4	4	3	0	27
Orthopedic	9	133.33 ± 47.69	3	6	14	3	9	7	8	0	50
Pediatric	4	70 ± 56.42	4	3	4	2	4	2	4	0	23
Plastic	4	101.25 ± 92.76	3	2	0	0	4	4	4	0	17
Thoracic	5	242 ± 112.33	5	0	7	2	5	5	5	0	29
Cardiovascular	9	253 ± 42.72	9	1	0	0	3	2	10	15	40
Maxillofacial	6	135 ± 77.65	1	6	8	2	5	5	7	0	34
**Technique**											
Open	56	164.73 ± 98.25	38	25	57	19	48	43	55	15	300
MIS^**a**^	18	80.83 ± 59.34	13	12	15	4	16	18	18	0	96
Combined^b^	2	202.5 ± 53.03	2	1	3	1	2	2	2	0	13
Total	76	145.85 ± 96.34	53	38	75	24	66	63	75	15	409

The number of samples by role in the table does not represent the number of unique participants. For example, an individual may have completed more than one questionnaire during different surgical procedures

^a^*MIS* minimally invasive surgery

^b^Combined: surgeries performed using both open and minimally invasive techniques

### Effect of the surgical time on workload

The average duration of each surgery was 145.85 ± 96.34 min. The minimally invasive technique showed a significantly shorter surgery duration compared to both the open technique and the combined technique (surgeries performed using both open and minimally invasive techniques, such as hysteroscopy with laparotomy) (*P* = 0.00). Although the combined technique had a shorter duration than the open technique, the difference was not statistically significant (*P* = 0.483). Moreover, the results of the Bonferroni test showed that the duration of surgery in the cardiovascular specialty was significantly longer compared to other surgical specialties (*P* = 0.00), except for neurosurgery and thoracic surgery. The Pearson correlation coefficient between surgical duration and all subscales of workload, except for the distraction and temporal demand subscales, demonstrated a statistically significant and positive relationship (Table 3). Table 3 displays the impact of various variables, such as surgical duration, surgical team roles, specialties, techniques, and job experience, on total workload and SURG-TLX subscales.

**Table 3:** *P* values for relationships between surgical variables, total workload, and SURG_TLX subscales

SURG-TLX subscales	Team role^a^	Surgery specialty^a^	Surgery technique^a^	Duration of surgery^b^	Job experience^c^
Mental demand	0.00	0.00	0.013	0.00 (0.308)	0.451 (*t* = 0.755)
Physical demand	0.00	0.00	0.00	0.00 (0.406)	0.785 (*t* = 0.274)
Temporal demand	0.005	0.00	0.475	0.308 (− 0.51)	**0.001 (*t *= 3.734)**
Task complexity	0.00	0.00	0.00	0.00 (0.422)	0.66 (*t* = − 0.44)
Situational stress	0.00	0.00	0.149	0.00 (0.333)	0.523 (*t* = 0.639)
Distraction	0.019	0.109	0.357	0.402 (− 0.042)	0.2 (*t* = 1.284)
Total workload	0.00	0.00	0.001	0.00 (0.367)	0.128 (*t* = 1.524)

Bold values indicate statistically significant result (*p* < 0.05)

^a^Separate univariate ANOVA

^b^Pearson correlation (*r*)

^c^Independent sample *t*-test (*t*)

### Effect of the job experience on workload

Three hundred forty-six (84.6%) of the participants had more than 2 years of job experience. The effect of job experience on perceived workload was analyzed with an independent sample *t*-test. The correlation between job experience and the subscales of Surge TLX was not statistically significant, except for temporal demands (Table 3).

### Effect of the surgical team role on workload

**Fig. 1:**
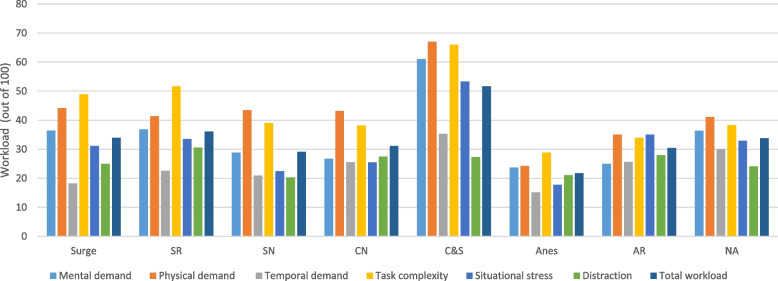
Average workload and SURG-TLX subscales for different surgical team roles

**Fig. 2:**
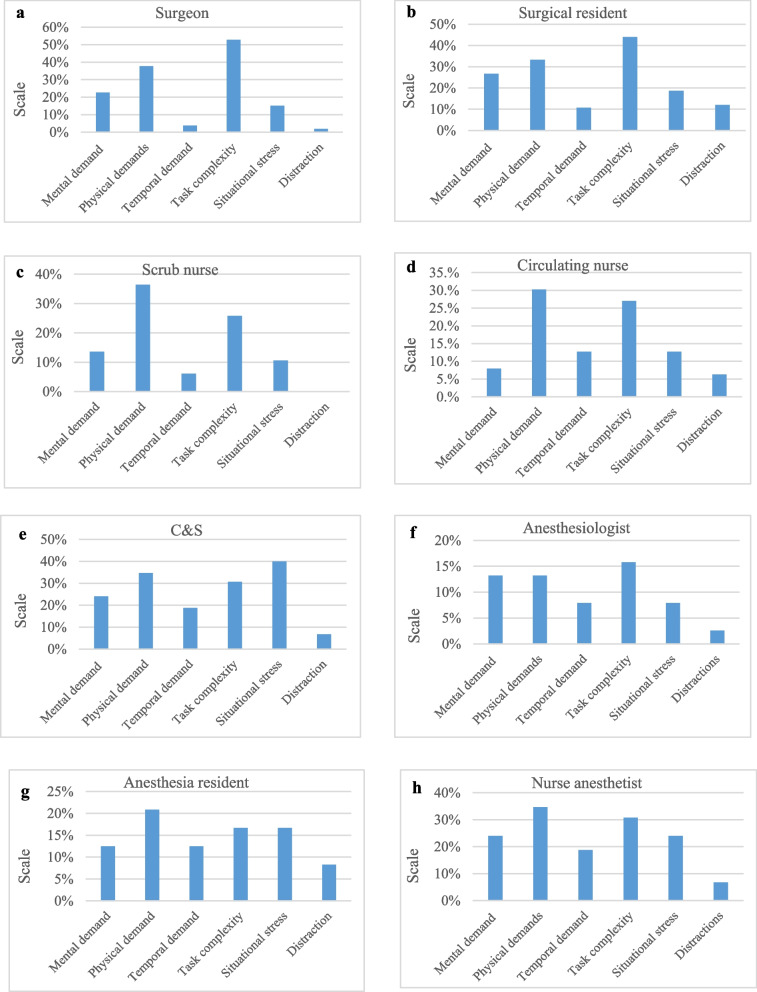
Percentage of cases above the midpoint (50) of SURG-TLX subscale by the role of surgical team members

The surgical team role was significantly associated with each of the SURG-TLX subscales (Table 3). The total workload among all participants was 32.41 ± 17.21. Surgical complexity, physical demands, and mental demands were the highest workload subscales, while distraction was the lowest. There was no statistically significant difference between the total workload of the surgeon and other members of the surgical team, except for the anesthesiologist (*p* = 0.016) and C&S (*p* = 0.008). C&Ss reported the highest mental demand, physical demand, time demand, surgical complexity, and situational stress, while anesthesia residents reported the highest distraction. Following C&Ss, surgical residents reported the highest mental demand and surgical complexity, whereas surgeons reported the highest physical demand. Additionally, following C&Ss, the anesthesia resident reported the highest stress and the anesthesia nurse reported the highest temporal demand. Anesthesiologists reported the least mental demand, physical demand, time demand, surgical complexity, and stress. Total workload and SURG-TLX subscales score for different surgical team roles are shown in Fig. 1 (page 21). Additionally, the percentage of cases exceeding the midpoint (50) for SURG-TLX subscales according to surgical team roles is shown in Fig. 2.

### Effect of surgical specialty on workload

**Fig. 3:**
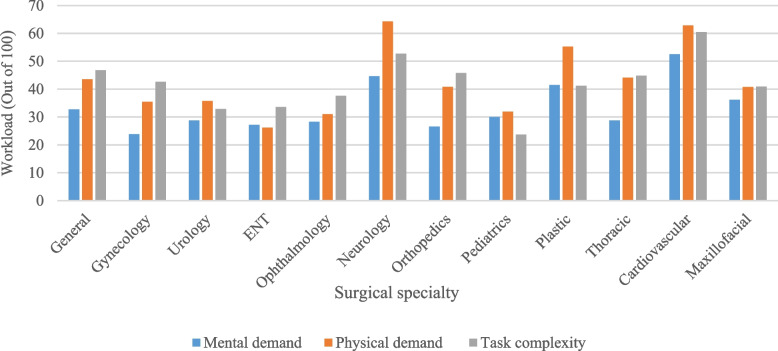
Three highest SURG-TLX subscales by surgical specialty

Surgical specialty had a significant effect on all workload subscales except the distraction subscale (Table 3). The surgical specialties with the highest total workload were cardiovascular (45.47 ± 15.68) and neurosurgery (38.88 ± 11.46). The lowest workload was also related to urology (25.93 ± 17.83) and ENT (26.69 ± 15.33). Figure 3 (page 23) demonstrates the 3 highest SURG-TLX subscales by surgical specialties. Examples of surgical procedures with high workload include pneumonectomy (55.33 ± 6.08), rhinoplasty (54.5 ± 12.5), humerus fracture (48.33 ± 15.78), gastric pull-up esophagectomy (46.94 ± 9.34), and coronary artery bypass grafting (CABG) (46.31 ± 16.08). Examples of surgical procedures with low workload include circumcision (8.66 ± 2.32), TURP: transurethral resection of the prostate (12 ± 4.91), and double-J stent removal (14 ± 5.74).

### Effect of surgical technique on workload

The surgical technique had a significant impact on the overall workload and the physical and mental demands experienced by the surgical team. Open surgery was associated with higher physical demands, while combined surgeries had the highest mental demands. In terms of surgical complexity, open surgeries were ranked highest, followed by combined surgeries and minimally invasive surgeries. Table 4 shows the average scores for each SURG-TLX subscale and the total workload across different surgical techniques. Open surgery was associated with significantly higher total workload (*P* = 0.001), physical (*P* = 0.00) and mental demands (*P* = 0.021), and surgical complexity (*P* = 0.00) compared to minimally invasive surgeries. However, there was no statistically significant difference observed in any of the subscales of the SURG-TLX between the open and combined surgical techniques.

**Table 4:** Average SURG_TLX scores by the different surgical techniques

Technique	Cases	Mental demand (Mean±sd)	Physical demand (Mean±sd)	Temporal demand (Mean±sd)	Task complexity (Mean±sd)	Situational stress (Mean±sd)	Distraction (Mean±sd)	Total workload (Mean±sd)
Open	300	34.46 ± 25.50	45.58 ± 26.89	24.31 ± 22.89	45.50 ± 26.46	30.86 ± 24.34	24.68 ± 16.61	34.23 ± 16.91
MIS	96	26.61 ± 22.63	28.17 ± 23.77	21.45 ± 22.05	31.61 ± 25.79	25.62 ± 27.14	26.82 ± 18.56	26.71 ± 17.28
Combined	13	40.76 ± 17.77	38.84 ± 23.81	20 ± 17.91	40.76 ± 28.27	24.23 ± 15.39	30 ± 24.57	32.43 ± 15.42
Total surgeries	409	32.82 ± 24.86	41.28 ± 27.06	23.50 ± 22.50	42.09 ± 26.94	29.42 ± 24.87	25.35 ± 17.37	32.41 ± 17.21

## Discussion

The results highlighted the impact of specialty, technique, and surgical role on workload in different healthcare centers. The average total workload score among participants was 32.41, which almost resembled the scores observed in previous studies conducted on the overall surgical team workload (Niichel et al. 2019; Cavuoto et al. 2017). Increased cognitive workload is a well-established phenomenon that has the potential to compromise surgical performance and increase the likelihood of surgical errors (Naik et al. 2022; Zhang et al. 2017).

This study confirms previous findings by demonstrating a significant increase in workload with the prolonged duration of surgery (Kennedy-Metz et al. 2020; Lowndes et al. 2020). The findings of the study conducted by Hallbeck et al. (2020) indicated a moderate correlation between surgical duration and the mental and physical demands of the surgeon (Hallbeck et al. 2020).

The highest sub-scales of workload were identified as surgical complexity, physical demands, and mental demands, respectively. In contrast to prior studies highlighting mental demands as the main source of high workload (Yu et al. 2016; Mazur et al. 2014; Lowndes et al. 2020) our study revealed higher scores for task complexity. This could indicate that the complexity of surgery is a considerable dimension for measuring the workload-specific to surgery.

The role of the surgical team had a significant effect on total workload, which is consistent with previous studies (Yu et al. 2016; Cavuoto et al. 2017; Zamudio et al. 2023). However, Lowndes et al.’s study showed that the surgical role only affects physical demand (Lowndes et al. 2019). In our study, perioperative nurses in the scrub and circulating roles (C&Ss) reported the highest workload across all subscales of the SURG-TLX, except for distraction. The demanding nature of their work in long-term cardiac surgeries, involving responsibilities, multitasking, and decision-making, likely contributed to their high workload. The results indicated that after C&Ss, surgical residents had the highest workload, followed by surgeons, anesthesia nurses, circulating nurses, anesthesia residents, scrub nurses, and anesthesiologists. After C&S, surgical residents had the highest mental demand and task complexity, whereas surgeons had the highest physical demand. Additionally, previous studies have identified that surgical residents have high mental demands (Lowndes et al. 2019; Yu et al. 2016) and surgeons have high physical demands (Yu et al. 2016) among various surgical teams. The study findings provide insights into the demands and responsibilities of each role, aiding in resource allocation and workflow optimization in surgical settings. There was no statistically significant difference between the total workload of the surgeon and other members of the surgical team, except for the anesthesiologist and C&S. This may indicate the presence of a shared mental model among the surgeon and other surgical team members. This mental model could be due to the fact that, in the hospitals we studied, members of the surgical teams in each specialty usually work together and remain consistent within their respective specialties, while having a high level of work experience (84.6% more than 2 years). Studies confirm that shared mental models enhance team communication and coordination, playing a crucial role in cognition, reasoning, and decision-making. They are vital for improving the safety and effectiveness of care (Wu 2018).

Surgical specialty had a significant effect on workload (Niichel et al. 2019; Zamudio et al. 2023). However, the results of some studies have shown that surgical specialty does not affect workload (Yu et al. 2016; Lowndes et al. 2020). Cardiovascular and neurology specialties had the highest workloads in our study (45.47 and 38.88, respectively). Previous research has shown that the total workload, measured by the SURG-TLX questionnaire, was 38.377 for the members of the cardiac surgery team after cardiovascular bypass (Kennedy-Metz et al. 2020), and 43.4 for the neurosurgery team (Bretonnier et al. 2020). Recent studies have brought to light the prevalence and effects of job burnout and workload among neurosurgeons (Jinli et al. 2019) and cardiothoracic surgeons (Sehgal et al. 2023; Bremner et al. 2022; Chow et al. 2021). Despite improving patients’ quality of life, cardiothoracic surgeons and neurosurgeons are susceptible to burnout, experiencing emotional, mental, and physical exhaustion which has consequences for surgeons, teams, and the healthcare system. However, the workload in these fields remains inadequately recognized and addressed (Sehgal et al. 2023; Zaed et al. 2020). Moreover according to Nischel et al. (Niichel et al. 2019), pediatric surgery was found to have the highest workload among eight surgical specialties. However, our study contradicts these findings, as we observed a lower workload in pediatrics compared to most specialties. This difference in workload could be influenced by factors such as the types and frequency of surgeries, duration of procedures, and working conditions.

The total workload and mental and physical demands were significantly higher in the open technique compared to the minimally invasive technique, which is consistent with the study conducted by Marçon et al. (Marçon et al. 2019). Although the results of some studies showed that the workload in laparoscopic surgery was higher than that in open surgery, the difference was not statistically significant (Yu et al. 2016; Law et al. 2020). A recent systematic review (2021) showed varied physical and mental outcomes among robotic, laparoscopic, and open surgical techniques due to methodological differences (Park et al. 2021). However, the high workload in the open surgery technique in the present study can be related to the complexity and long duration of the open surgeries.

In this study, none of the participants reported psychotropic medication use and only one surgical team member was excluded due to visible anxiety symptoms. Noteworthy research suggests that healthcare professionals, particularly surgeons, tend to underreport mental illness or psychotropic medication use due to associated stigma (Fu et al. 2021; Aggarwal et al. 2020; Clare and Richard 2014). Importantly, our inclusion criterion aimed to control potential confounding variables for the validity of our findings, not to perpetuate discrimination.

## Limitations

Despite the high sample size in the present study, the number of samples could have been even higher as our study was conducted in multiple centers. Due to significant differences in the surgical procedures performed in open and minimally invasive techniques, determining which technique has a higher workload is challenging. Additionally, shifts, duration of shifts, and hours worked per day or week for each surgical team member were not investigated in our study.

## Conclusion

The results of the study showed that the workload for some members of the surgical team is disproportionately high and is influenced by factors such as specialty, technique, role, and surgical duration. By knowing the distribution of workload among the members of the surgical team, efforts can be made to optimize the team members' workload and reduce the three high workload subscales (task complexity, mental demands, and physical demands) in C&Ss, cardiovascular specialties, open surgical techniques, and long-duration surgeries.

## Data Availability

The dataset is potentially available upon request.
